# Microgreens: Functional Food with Antiproliferative Cancer Properties Influenced by Light

**DOI:** 10.3390/foods10081690

**Published:** 2021-07-22

**Authors:** Francesca Truzzi, Anne Whittaker, Chiara Roncuzzi, Annalisa Saltari, Mitchell P. Levesque, Giovanni Dinelli

**Affiliations:** 1 Department of Agricultural and Food Sciences, University of Bologna, Viale Fanin, 44-40127 Bologna, Italy; whittaker.anne@gmail.com (A.W.); chiara.roncuzzi3@studio.unibo.it (C.R.); giovanni.dinelli@unibo.it (G.D.); 2 Department of Dermatology, University of Zurich Hospital, University of Zurich, Zurich, CH 8952 Schlieren, Switzerland; annalisa.saltari@usz.ch (A.S.); mitchell.levesque@usz.ch (M.P.L.)

**Keywords:** microgreens, green pea, Red Rambo radish, LED, Ewing sarcoma, RD-ES, A673, polyphenols

## Abstract

The anti-proliferative/pro-oxidant efficacy of green pea, soybean, radish, Red Rambo radish, and rocket microgreens, cultivated under either fluorescent lighting (predominant spectral peaks in green and orange) or combination light-emitting diode (LED, predominant spectral peak in blue) was investigated using Ewing sarcoma lines, RD-ES and A673, respectively. All aqueous microgreen extracts significantly reduced cell proliferation (cancer prevention effect) to varying extents in two-dimensional sarcoma cell cultures. The effect of the polyphenol fraction in the aqueous food matrix was unrelated to total polyphenol content, which differed between species and light treatment. Only *Pisum sativum* (LED-grown) extracts exercised anti-proliferative and pro-apoptotic effects in both three-dimensional RD-ES and A673 spheroids (early tumor progression prevention), without cytotoxic effects on healthy L929 fibroblasts. A similar anti-tumor effect of Red Rambo radish (LED and fluorescent-grown) was evident only in the RD-ES spheroids. Aside from the promising anti-tumor potential of the polyphenol fraction of green pea microgreens, the latter also displayed favorable growth quality parameters, along with radish, under both light treatments over the 10 day cultivation period.

## 1. Introduction

Microgreens, an emerging specialty crop for the 21st Century, are tender, immature vegetable greens produced from the seeds of vegetables, herbs, and grains, including wild species with delicate textures and distinctive flavors [1,2]. Composed of a central stem, cotyledon leaf/leaves and a pair of young true leaves, microgreens are generally harvested between 10 and 14 days from seeding [2]. Aside from advantages pertaining to environmental sustainability and adaptation to increased urbanization and global climate change, microgreen cultivation is of great interest from the standpoint of promoting human health [2,3]. Microgreens are shown to contain significantly higher contents of mineral elements and phytochemical constituents (alkaloids, various terpenoids, and polyphenols) than their mature leaf counterparts [1,2,3]. Based on studies conducted on both the sprouts and mature leaf counterparts of various microgreens such as the *Brassicaceae*, these bioactive phytochemicals are reported to be of pharmaceutical importance, attributable to antioxidant, anti-inflammatory, and anti-cancer properties [4,5,6]. The phytochemical composition of microgreens is influenced by both cultivar selection and growing conditions [2,7,8]. Microgreens are predominantly an indoor production, cultivated either on soil or hydroponically. The recent introduction of light-emitting diode (LED) lights in controlled-environment agriculture has provided incentive towards investigating the modulatory effects of light spectral qualities on the biosynthesis of phytochemicals in microgreens with known antioxidant and anti-inflammatory effects [7,9,10,11,12,13]. From experiments investigating the effect of conventional white (W), monochromic blue (B), or red (R) LED light, as well as various percentage combinations of B, R, green (G), amber (A), and far-R LED, the results show both species- and light-dependent responses in the expression of antioxidant activity and polyphenol, carotenoid, and chlorophyll contents, respectively. Although distinct variations are noted for individual polyphenol constituents and different species, total polyphenol expression is favored by B LED light compared to W and R LED light [7,10,12] and by combined B and A LED [11]. Given that the use of LEDs for microgreen production to enhance phytochemical content is a relatively new area of research, further studies are needed to ascertain suitable lighting on a species-by-species basis [11].

Despite being reputed as promising in anti-cancer prevention [2], the “functional food” potential of microgreens, determined by the capacity to improve/regulate a specific metabolic process/mechanism towards either preventing or controlling a disease [14], was only recently reported for the first time [15]. Given that cancer is the second leading cause of death globally and is predicted to increase over the coming decades, especially in low- and middle-income countries [16], there is an urgent requirement for cost-effective cancer prevention measures by increasing the intake of bioactive plant-derived phytochemicals [16,17]. De la Fuente et al. (2020) [15] evaluated the effect of the bio-accessible fractions extracted from four *Brassicaceae* microgreens (broccoli, kale, mustard, and radish) on human colorectal adenocarcinoma cells (Caco-2). Those authors reported an anti-proliferative effect on the cells, increases in reactive oxygen species (ROS), and general cell cycle arrest in G2/M, as well as apoptotic cell death. It was proposed that the daily intake of microgreens within a balanced diet could be a preventive (preclinical) nutritional strategy to reduce the burden of colon cancer [15]. Both sprouts and microgreens are suggested to provide horizons for novel research, meriting special attention in multiple avenues, including drug discovery [18].

The transformation of normal cells into malignant cells is multi-step process, involving genetic alterations including mutations, amplifications or deletions, and/or epigenetic changes [16]. Cancer cells aggregate into three-dimensional (3D) avascular, solid tumor, spheroid structures. These structures are comprised of an external layer of cells displaying high proliferation rates, a middle layer of senescent cells, and a central core of necrotic cells, respectively [19]. The promotion stage of cancer development is a lengthy process and appears to be reversible with intervention by preventive drugs/agents, indicating that the initiation and promotion stages should be the preferential choice for prevention strategies [16]. Various phytochemical constituents are reputed to be effective cancer preventive agents in this preclinical stage, as well effective cancer adjuvants in clinical treatment aimed at reducing tumor progression. The efficacy of plant-derived bioactive phytochemicals would necessitate a pro-oxidant strategy in promoting apoptosis, cell cycle arrest and the inhibition of various signal transduction pathways involved in cancer pathogenesis, as well as a complementary and/or synergistic action with chemotherapeutic agents in clinical settings [14,16,17,20].

Given the anti-proliferative effect of *Brassicaceae* microgreens on Caco-2 cell lines [15] and the need for future work on microgreens to further test the health-promoting functional effects [18], as well as to investigate LED lighting on the health-promoting phytochemical content [2], the present study was directed at addressing these aspects. More specifically, the aim of the study was to investigate the functional efficacy of the polyphenol fraction within the food matrix, extracted from five microgreen species grown under LED lighting, in reducing in vitro tumor proliferation. To this end, the anti-proliferative/pro-oxidant efficacy of two *Fabaceae* (pea and soya) and three *Brassicaceae* (rocket (arugula), radish, and the Red Rambo radish) microgreens, cultivated under either fluorescent or LED (spectral light peaks B:G:R with predominance in B) light, was investigated using 2D, as well as 3D homotypic Ewing sarcoma (ES) spheroid lines (rhabdomyosarcoma (RD-ES) and A673, respectively). The use of 3D spheroids is predicted to be the gold-standard in vitro model for investigating therapeutic agents in cancer research [21]. To date, the use of 3D spheroids for the screening of plant extracts and individual dietary phytochemicals is an emerging field of research. The present paper reports for the first time the effects of microgreen extracts on spheroid cultures, which more accurately mimic some features of solid tumors such as spatial architecture, physiological responses, gene expression patterns, and drug resistance mechanisms [19,21].

## 2. Materials and Methods

### 2.1. Plant Material and Growth Conditions

The five microgreen species considered were: green pea (*Pisum sativum*, *Fabaceae*), radish (*Raphanus sativus*, *Brassicaceae*), Red Rambo radish (*Raphanus sativus*, *Brassicaceae*) soybean (*Glycine max*, *Fabaceae*), and rocket (also called arugula, *Eruca vesicaria* subsp. *Sativa*, *Brassicaceae*).

The two *Fabaceae* species were pre-soaked for 24 h to accelerate germination. All species (25 seeds per replicate) were germinated in circular pots with a diameter of 10 cm for green pea and 8 cm for the remaining species, respectively. Seeds were placed on the surface of a neutral peat moss (*Sphagnum*) expanded clay mixture in a ratio of 80:20%, and dark incubated for 24 h at 18 °C. After germination, germinated seeds were then subjected to the respective fluorescent (control) and LED light treatments. For each species and light treatment, there were 5 replicates for each time point after sowing at 1, 2, 7, and 10 days (final harvest), respectively.

Both the fluorescent and LED lighting were positioned at a distance of 60 cm above the germinated seeds. Microgreens were cultivated at 18 °C with a photoperiod of 16/8 h (light/dark). For the control treatment, fluorescent tube lighting (neon, FH21830, 21 W, warm white 830, 85 cm, OSRAM SpA, Milan, Italy) was used. From a spectrometric analysis (Miniature Fibre Optic, USB2000+UV-VIS, Ocean Optics, Milan, Italy), light quality was characterized by two major spectral profile peaks in G and orange and a minor peak in B in the Supplementary Figure S1. For the LED treatment, LED tube lighting (Top light, Natural Indoor Plus, 230 V, 75 W, C-LED, Imola, Italy) with a major spectral profile peak in B and additional spectral peaks in G and R (Figure S1) was used. For the fluorescent lighting, each replicate treatment of 5 pots was exposed to an average light intensity (portable Luxmeter, Delta OHM, Padova, Italy) of 65 μmol m^−2^ s^−1^ photosynthetic photon flux density (PPFD) at canopy level. Similarly, for the LED lighting, with uniformity of the light environment, each replicate treatment of 5 pots was exposed to an average of 270 μmol m^−2^ s^−1^ PPFD at canopy level. Microgreens were watered daily with distilled water.

At each of the time points after sowing (1, 2, 7, and 10 days), fresh samples were used for the growth parameter measurements. A portion of each of the fresh samples was frozen in liquid nitrogen and stored at −80 °C for the phytochemical analyses. All growth parameters and phytochemical analyses were conducted in triplicate.

### 2.2. Measurement of Growth Parameters

For the analysis of microgreen growth, the following were considered: ground cover, hypocotyl length, fresh weight, and dry weight. To estimate ground cover, photographs were taken from above each pot from a set position with a metric reference to define the scale of the image. The photographs were analyzed with ImageJ (Wayne Rasband, NIH, Bethesda, MD, USA). The pot area and microgreen surface area, respectively, were measured. Ground cover was calculated as follows: (microgreen area/pot area) × 100. Hypocotyl length was then measured on 4 random plants within each of 5 replicate pots. Individual plants for each treatment were pooled, washed, and the roots excised. The microgreen plantlets were blotted dry and weighed (fresh weight) and then dried in an oven (dry weight) at 4 h at 70 °C.

From the growth parameters (dry weight, leaf area, and pot area), the relative growth rate (RGR), leaf area index (LAI), and net assimilation rate (NAR) were calculated. The RGR was calculated as the dry matter accumulation from the leaf area growth per day. The LAI was the leaf area/surface area, and the NAR was calculated from RGR/LAI.

### 2.3. Phytochemical Analyses

Liquid nitrogen-frozen tissues were homogenized with a pestle in a pre-cooled motor. Distilled water in a volume/tissue ratio of 2/1 was then added to ground tissue samples on ice to extract the total polyphenol content. Extracts were agitated for 30 min to facilitate extraction, centrifuged, and filtered (45 µM). The supernatants were used for the measurement of polyphenol content and ferric reducing antioxidant potential (FRAP). Polyphenols were measured according to the Folin–Ciocalteau spectrophotometric (765 nm) method using gallic acid as a reference standard [22]. FRAP (reduction of Fe^2+^) was determined using a spectrophotometric (593 nm) method reported previously [23].

For the extraction and measurement of the carotenoids and chlorophyll, the method of Porra et al. (1989) [24] was used. Liquid-nitrogen-frozen tissues (0.15–0.5 g) were homogenized on ice with a motor and pestle in the dark, and tissues extracted in 500 µL 80% acetone three times until the pellet was devoid of color. Supernatants were pooled and used to measure chlorophyll and carotenoids, according to equations (1) to (4) as follows:

Chlorophyll a: 12.25 × absorbance at 663 nm − 2.55 × absorbance at 648 nm,(1)

Chlorophyll b: 20.31 × absorbance at 648 nm − 4.91 × absorbance at 663 nm,(2)

Chlorophyll a + b: 17.76 × absorbance at 648 nm + 7.34 × absorbance at 663 nm,(3)

Carotenoids: (1000 × absorbance at 470 nm − 1.82 × (Chlorophyll a) − 85.02 × (Chlorophyll b))/198(4)

Aqueous extracts, from which polyphenol content was measured, were used to investigate effects on healthy fibroblasts and the sarcoma cell lines. To ensure the removal of micro-organisms, the plant extracts were autoclaved for 15 min at 121 °C [25].

### 2.4. Experimentation with Cell Lines

#### 2.4.1. Culture Conditions

L929 mouse fibroblasts (ATCC-CCL1, ATCC, Manassas, VA, USA) were cultured with Dulbecco’s modified Eagle medium (DMEM), to which 10% fetal bovine serum, 1 mM L-glutamine, and 1% penicillin–streptomycin were added. Ewing sarcoma (ES) cell lines (rhabdomyosarcoma (RD-ES) and A673) respectively, provided by Watchel (University of Zurich, University of Zurich Hospital, University of Zurich, Zurich, Switzerland), were cultured in Roswell Park Memorial Institute (RPMI)-1640 medium, supplemented with 10% FBS (fetal bovine serum), 2% glutamine, 1% sodium pyruvate, and 1% penicillin–streptomycin. Stock cultures of all cell lines were maintained at 37 °C in a humidified atmosphere containing 5% CO_2_ in tissue culture flasks (75 cm^2^; BD Biosciences), and the culture medium was changed every two days. Prior to experimentation, the cells were trypsinized and cell density evaluated microscopically using a Bürker counting chamber.

#### 2.4.2. Generation of 3D Sarcoma Spheroids

To obtain 3D multicellular spheroids, the liquid overlay method was used [26]. Tissue culture (96-well) plates were coated with 100 μL 1.5% agar dissolved in sarcoma base medium (RPMI). Polymerized agar was irradiated with UVB for 30 min. Limitations with the liquid-overlay technique to generate spheroids include the formation of unequal-size spheroids and in certain cell lines, the inability to form compact spheroids [27]. For this reason, it was important to investigate the optimum cell number for spheroid formation along with the associated morphological parameters. RD-ES cells (2500, 5000, 10,000) were seeded in each well (80 µL volume) and spheroid formation was monitored and photographed 24, 48, 96, and 144 h after plating. Pictures were analyzed and spheroid area and diameter calculated using ImageJ (Wayne Rasband, NIH, Bethesda, MD, USA) software.

RD-ES cells were shown to form compact spheroid structures with a round geometry, and not-loose aggregates, within 24 h (Figure S2). Spheroids displayed a stratification gradient with a well-defined dark necrotic central region, evident after 48 h in spheroids generated from 5000 cells (Figure S2). Spheroid area and diameter were homogenous, with variation accounting for 5% and less at the selected time points (Table S1). Intermediate-size spheroids were provided using 5000 cells (Table S1), and for this reason, this number was selected for spheroid formation in all subsequent experiments. It was also the number selected for experiments on A673 cells (Table S1).

#### 2.4.3. Cell Proliferation Measurements in 2D Cell Lines and 3D Spheroids

L929, RD-ES, and A673 cells were plated onto 96-well tissue culture plates (10,000 cells/well) in complete medium. After 24 h at 37 °C, cell lines were treated with microgreen extracts (20 or 40 µL) in the same volume of DMEM (L929 cells) or RPMI-1640 (sarcoma lines), respectively, or with water as the control (suspended in DMEM or RPMI alone). Cells were incubated with the extracts for a further 24 h. Cell proliferation in L929, A673, and RD-ES after exposure to the microgreen extracts was then detected using the 3-(4,5-dimetiltiazol-2-il)-2,5-difeniltetrazolio (MTT) assay, according to the ISO 10993-5 International Standard procedure [28]. The method is based on the reduction of MTT by mitochondrial dehydrogenase of intact cells to produce purple formazan, determined by measuring the absorbance at 540 nm using a multi-well scanning spectrophotometer (Labsystems Multiskan MS Plate Reader, ThermoFisher Scientific), as described by Truzzi et al. (2020) [29].

For the MTT assay on spheroids, cells (5000 cells/well) were seeded onto 96-well culture plates in complete medium. After 24 h at 37 °C, cell lines were treated with microgreen extracts as described above for the 2D cultures for a further 24 h. The spheroids were incubated with 0.5% MTT for 4 h at 37 °C and then dissolved with 100 μL isopropanol with 0.04 N HCl. Before analysis, disaggregated spheroids were transferred into empty wells. The plate was read at 560 nm with a reference filter of 650 nm [30].

Six replicates were performed for each microgreen extract obtained from the respective plants cultivated under both fluorescent and LED light treatments. All results from the MTT assay were expressed as a viability percentage, compared to the control.

#### 2.4.4. Spheroid Area and Necrotic Region Area Measurements of 3D Spheroids

Sarcoma cells (5000 cells/well) were seeded onto 96-well culture plates in complete medium. After 24 h at 37 °C, cell lines were treated with microgreen extracts (20 or 40 µL) in RPMI-1640 in a total volume of 80 µL, or with water as the control (suspended in RPMI medium alone). Prior to adding the microgreen extracts to spheroids, and after 24, 48, and 120 h incubation at 37 °C, the spheroids were examined under an optical microscope (Eclipse Ts2, Nikon) at 10 times magnification and photographed.

The photographs were examined using ImageJ software and the images processed to pixels (300 pixels/2.54 cm) as described by Saltari et al. (2016) [30]. To estimate the anti-proliferative effect, total spheroid area was calculated. The pro-apoptotic effect was measured by calculating the size area of the central necrotic zone. At 24 h from spheroid formation (time zero), the respective spheroid and necrotic area zones of all spheroids were calculated, just prior to exposure to each of the respective microgreen extracts (20 and 40 µL) for 24, 48, and 120 h. After each time point, the spheroid areas and necrotic zones were expressed as percentage relative to time zero, respectively, and then expressed as a percentage of the untreated control. This approach was designed to overcome any disparity attributable to differences in spheroid areas, even if, generally, the spheroids were largely homogenous (Table S1).

### 2.5. Statistical Analysis

Statistical analysis was conducted using CoStat version 6.450 (2017) software (http://www.cohort.com). Significance was determined by one-way variance (ANOVA) and the Tukey–Kramer test to identify any significant differences between treatments at *p* ≤ 0.05. Two-way variance was conducted for the interactions between light treatment and plant species.

## 3. Results

### 3.1. Growth Quality Parameters in Microgreens

**Figure 1:**
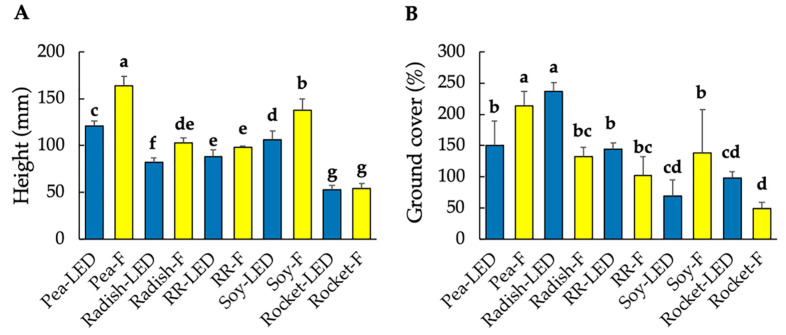
Plant height (**A**) and ground cover (**B**) of green pea, radish, Red Rambo (RR), soybean (soy), and rocket microgreens, cultivated under LED and fluorescent (F) lighting. The different lowercase letters (a–g) represent significant differences between the microgreen species under the different lighting for height and ground cover (*p* < 0.05), as determined from a one-way ANOVA.

Fresh weight is an important growth quality parameter of microgreens [13], and fresh weight accumulation is assessed by growth parameter measurements (including RGR, LAI, and NAR). Significant differences in the overall fresh weight (and resultant dry matter) between species were species dependent and not light-treatment dependent (Table 1). Green pea, followed by radish, accumulated the highest fresh mass over the 10 day period (Table 1), and this was also reflected in the higher ground cover for the latter two species. As with fresh mass, light treatment had no impact on overall ground cover, although the interaction between species and light treatment was significant (Table 1). Whereas for green pea, ground cover was significantly higher under fluorescent lighting, for radish, significantly higher ground cover was evident under LED lighting (Figure 1B). Additionally, hypocotyl length was notably higher for green pea than the remaining microgreens. Although fluorescent light impacted more significantly than LED on overall hypocotyl length (Table 1), the significant interaction for species and light treatment (Figure 1A) indicated that for Red Rambo radish and rocket, lighting treatment had no impact on hypocotyl length.

**Figure 2:**
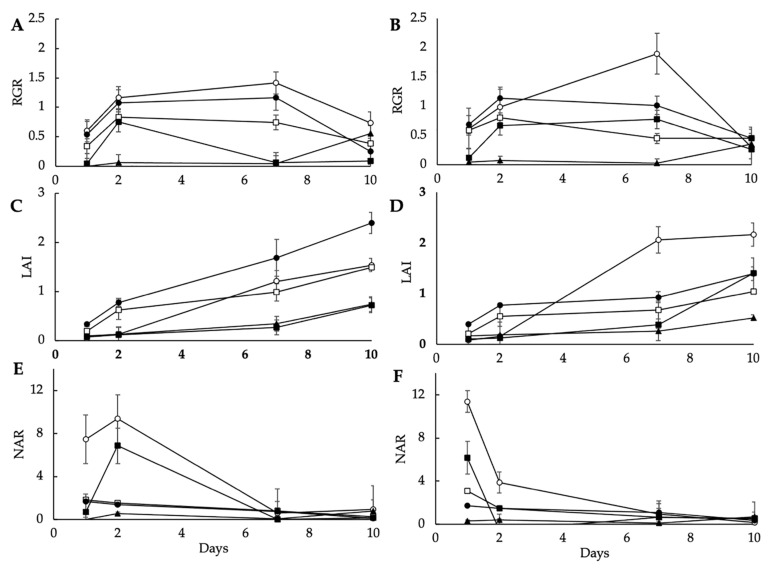
Relative growth rate (RGR: g/m^2^ surface area/day) under LED (**A**) and fluorescent lighting (**B**), leaf area index (LAI: m^2^ leaf area/m^2^ surface area) under LED (**C**) and fluorescent lighting (**D**), and net assimilation rate (NAR: g/m^2^/day) under LED (**E**) and fluorescent lighting (**F**), of green pea (○), radish (●), Red Rambo (RR, □), soybean (soy, ■ ), and rocket (▲) microgreens over a 10 day period. Values are the mean ± SD of three replicates (*n* = 3).

**Figure 3:**
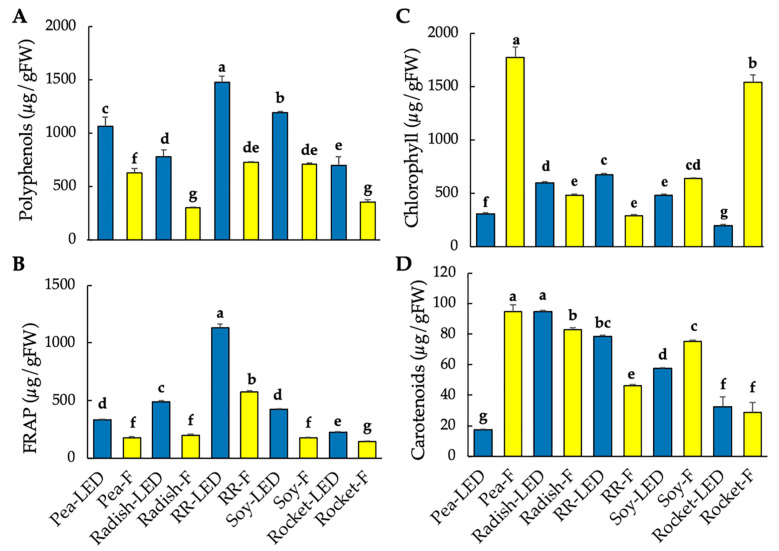
Polyphenol content (**A**), FRAP (**B**), chlorophyll ((**C**), sum total of chlorophyll a and b), and carotenoid (**D**) content of green pea, radish, Red Rambo (RR), soybean (soy), and rocket microgreens, cultivated under LED and fluorescent (F) lighting for 10 days. The different lowercase letters (a–g) in each graph denotes significant differences between the microgreen species under different lighting at *p* < 0.05, as determined from a one-way ANOVA.

Similar to fresh weight, dry weight, and ground cover after the 10 day period, the average measurements for RGR, LAI, and NAR, respectively, over the 10 day period were species dependent (Table 2). There were no overall significant differences between the two light treatments or the interaction between species and light treatment (Table 2). Examining the trends over time (Figure 2), the significantly higher accumulation of fresh mass in green pea (Table 1) was evidenced by a higher RGR until day 7 (Figure 2A,B) and higher initial NAR values (Figure 2E,F). The higher overall ground cover under fluorescent lighting (Figure 1B) for green pea was reflected by the more rapid and higher LAI under fluorescent lighting compared to that of the LED (Figure 3C,D). The converse was evident for radish, in which there was increased ground cover under LED lighting (Figure 1B) and a more rapid and higher LAI under LED compared to that of the fluorescent lighting (Figure 2C,D). In addition to green pea, the present conditions were also optimal for the growth of radish (Figure 2). In contrast, the cultivation environment was not optimal for rocket in terms of the duration of growth. Rocket microgreens are generally reported to be slow growing (15–25 days), and this was evident from the RGR, LAI, and NAR measurements over time (Figure 2) and the low fresh weight accumulation (Table 1).

### 3.2. Phytochemical Content in Microgreens

Given that the use of LEDs for microgreen production to enhance phytochemical content for disease prevention is a relatively new area of research [2] and that polyphenols, specifically, are considered an important quality [13], total polyphenols and FRAP were measured along with carotenoid and chlorophyll content. Both polyphenol content and FRAP were significantly higher under LED lighting, whereas fluorescent lighting favored a higher production of chlorophyll and carotenoids (Table 3). Highly significant differences were reported between species, as well as the interaction between species and light treatment (Table 3). Red Rambo radish displayed both the highest polyphenol content and FRAP, particularly under LED (Table 3, Figure 3A,B). The lowest polyphenol content and FRAP were evident in rocket (Table 3, Figure 3A,B). Chlorophyll content was significantly higher in green pea, followed by rocket, under fluorescent lighting (Figure 3C). Interestingly, higher levels of chlorophyll and carotenoids were shown for the two radish species under LED lighting (Figure 3C). The highest overall carotenoid content was shown for green pea (fluorescent-grown) and radish (LED-grown), respectively.

### 3.3. Polyphenol Content in Microgreen Extract Matrix Administered to Cell Lines

Given the time duration required to test the effects of microgreen extracts on spheroid cultures, it was important to sterilize the extracts to ensure the removal of all microbiota. The aqueous extraction precluded the presence of carotenoids and chlorophyll as potential functional phytochemicals administered to the cells. Hence, the premise was that any potential anti-cancer effects would largely be attributable to the presence of polyphenols, unaffected following autoclave sterilization (Table 4) as reported previously [25]. An extract volume of 20 µL for all microgreens was administered to the cell lines. An approximate concentration of 5 µg polyphenols was previously shown to not be cytotoxic to Caco-2 cell lines [29]. Although the polyphenol content in the 20 µL volume of all microgreen extracts investigated was lower than 5 µg (Table 4), this volume was selected as polyphenol content was within a “food extract” matrix, that had not previously been tested for cytotoxic effects. Within the 20 µL volumes, the polyphenol contents varied from 0.32 to 1.43 µg/gFW depending on both lighting and the microgreen species used (Table 4). The highest polyphenol contents within the 20 µL volumes were evident for all microgreens cultivated under LED lighting, with Red Rambo containing the highest content, followed by soybean, green pea, rocket, and radish in descending order, respectively (Table 4). The polyphenol content under fluorescent lighting was 64, 44, 56, 67, and 44% of that under LED lighting for green pea, radish, Red Rambo, soybean, and rocket, respectively (Table 4).

#### 3.3.1. Anti-Proliferative and Pro-Apoptotic Effects of Microgreen Extracts on RD-ES Sarcoma Cells in 2D Culture and in 3D Spheroids

**Figure 4:**
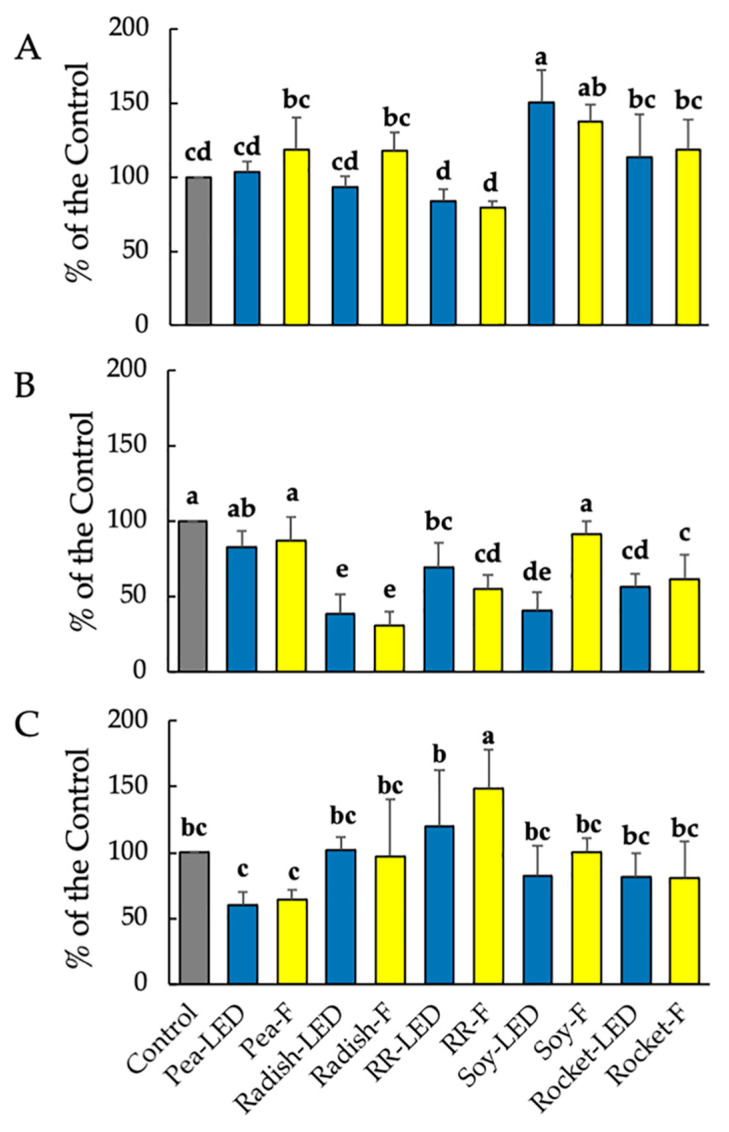
Cell vitality (MTT assay) on healthy L929 fibroblasts (**A**), 2D RD-ES (**B**), and 3D RD-ES (**C**) exposed for 24 h to 20 µL green pea, radish, Red Rambo (RR), soybean (soy), and rocket microgreen extracts, cultivated under LED and fluorescent (F) lighting. Values are the mean ± SD of six replicates (*n* = 6). The different lowercase letters (a–e) within each graph represent significant differences between the microgreens under different lighting for each treatment (*p* < 0.05), as determined from a one-way ANOVA.

The MTT assay is one of the most widely used methods for cytotoxicity screening of compounds on cancer cells [31]. As a control, selected concentrations of compounds are, similarly, tested on healthy cell lines. Fibroblast L929 cells were used for this purpose, according to the UNI EN ISO 10993/2009 ruling. Aqueous microgreen extracts of 20 µL were firstly examined. Relative to the control containing no added plant extract, all microgreen plant extracts investigated in the present study were not cytotoxic to healthy cells, and cell proliferation of the L929 fibroblasts was not significantly decreased after 24 h compared to the control (Figure 4A). Given that 2D cancer cell models have been and are still widely used for compound screening [32], the effect of 20 µL plant extracts was then tested on 2D RD-ES cells. With the exception of green pea (LED and F) and soy (F), the remaining microgreen extracts significantly reduced cell proliferation of the RD-ES cells after 24 h compared to the control (Figure 4B). This was particularly evident for radish (LED and F) and soy (LED) (Figure 4C). In contrast to the 2D RD-ES, when the RD-ES were cultivated in tumor form (3D RD-ES), no significant effect on cell vitality was shown compared to the untreated control exposure for 24 h to the microgreen extracts (Figure 4C). Given the more complex structural morphology of spheroid cultures, longer exposure times (96 h) to drug compounds have been implemented prior to MTT testing [31]. In the present investigation, the compact aggregates of the RD-ES were not amenable to MTT testing after 96 h.

**Figure 5:**
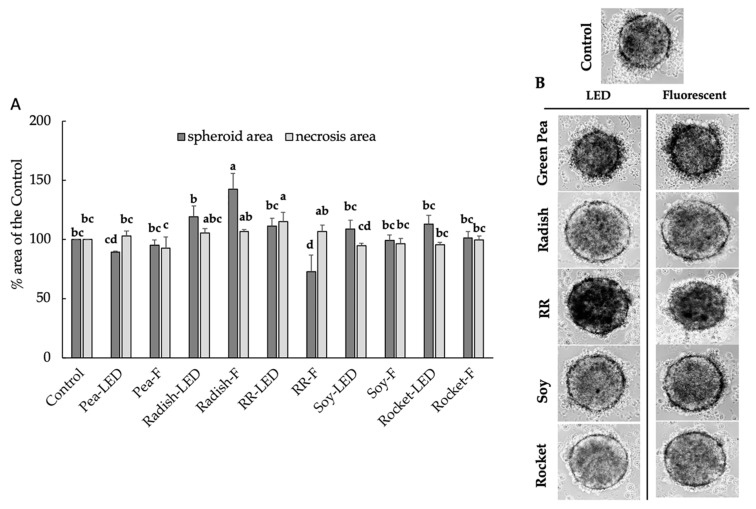
The anti-proliferative (spheroid area) and pro-apoptotic effects (necrosis area) (**A**) on RD-ES exposed for 120 h to 20 µL green pea, radish, Red Rambo (RR), soybean (soy), and rocket microgreens, cultivated under LED and fluorescent (F) lighting. Values are the mean ± SD of six replicates (*n* = 6). The different lowercase letters (a–d) represent significant differences for the microgreen species under different lighting for spheroid area and necrosis area, respectively (*p* < 0.05), as determined from a one-way ANOVA. Associated images showing pro-apoptotic effects (necrosis area) of RD-ES are shown (**B**).

Despite the more complex structural morphology, spheroid cultures are physiologically more relevant to study tumors, and the anti-proliferative and pro-apoptotic effects of the microgreen extracts on RD-ES were examined [19]. The anti-proliferative effect involved calculating total spheroid area, indicative of cytotoxic effects on the outer proliferative zone, whereas the pro-apoptotic effect was measured by the area of the central necrotic zone. After exposure for 24 and 48 h, no effects were evident (results not shown). After 120 h exposure to the plant extracts, there was a significant anti-proliferative effect by Red Rambo (F) shown by a significant decrease in spheroid area. Moreover, there was a significant pro-apoptotic effect demonstrated by the increase in the necrotic area of Red Rambo (LED) and to some extent Red Rambo (F) (Figure 5A,B). The remaining microgreen extracts produced no effect. Despite the significant protective effect of radish and rocket on the 2D RD-ES (Figure 4B), no anti-tumor effect was evident for the 20 µL volume on RD-ES in 3D spheroid formation after 120 h (Figure 5A,B).

**Figure 6:**
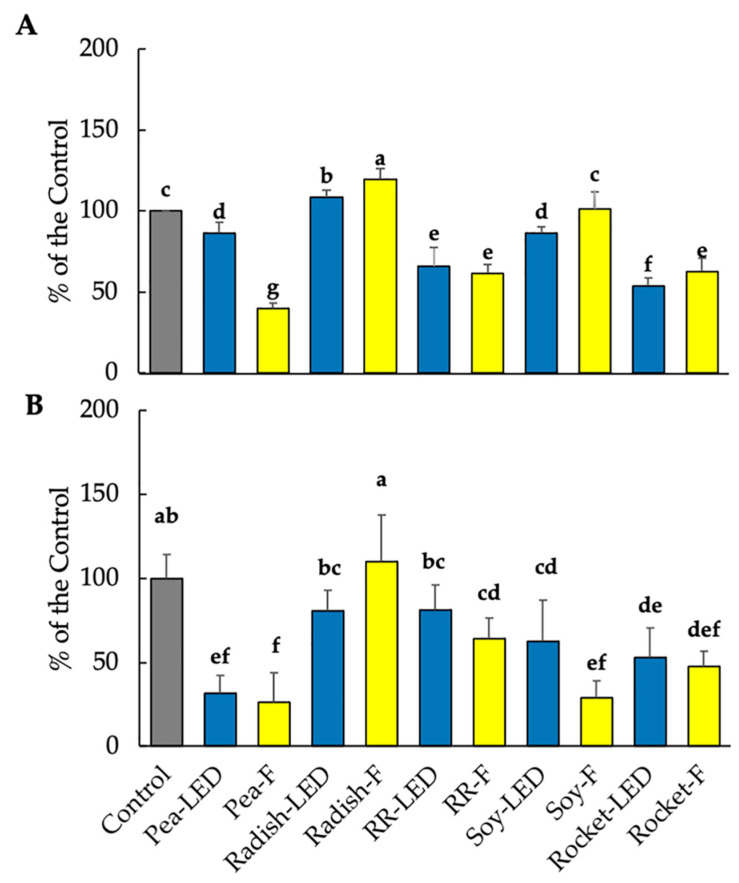
Cell vitality (MTT assay) on healthy L929 fibroblasts (**A**), and 3D RD-ES (**B)** exposed for 24 h to 40 µL green pea, radish, Red Rambo (RR), soybean (soy), and rocket microgreen extracts, cultivated under LED and fluorescent (F) lighting. Values are the mean ± SD of six replicates (*n* = 6). The different lowercase letters (a–f) represent significant differences in the microgreen species under different lighting for each treatment (*p* < 0.05), as determined from a one-way ANOVA.

The microgreen extract volume was then increased to 40 µL to investigate whether the effects were concentration dependent. As with the 20 µL volume, cytotoxicity using the MTT assay was examined on both healthy L929 fibroblasts and 3D RD-ES spheroids after 24 h (Figure 3B and Figure 6A). The 40 µL volume was shown to be approaching toxic effects on healthy L929 cells. A significant toxic effect on L929 fibroblast cell vitality was evident following exposure to green pea (F), Red Rambo (LED and F), and rocket (LED and F) with an average decrease in proliferation of approximately 60, 37, and 42% relative to the control, respectively (Figure 6A). Notably, in contrast to the 20 µL volume, a cytotoxic effect was noted after only 24 h on RD-ES spheroids of garden pea (LED and F), RR (F), and rocket (LED and F), amounting to an average of 82, 34, and 50% of the control, respectively (Figure 6B).

**Figure 7:**
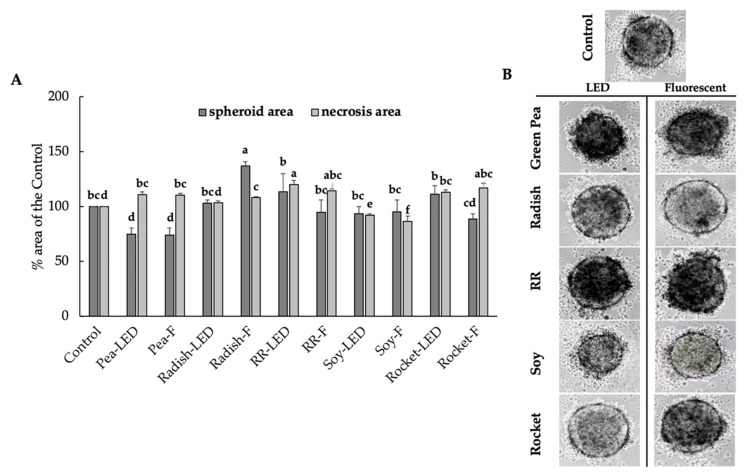
The anti-proliferative (spheroid area) and pro-apoptotic effects (necrosis area) (**A**) on RD-ES exposed for 120 h to 40 µL green pea, radish, Red Rambo (RR), soybean (soy), and rocket microgreens, cultivated under LED and fluorescent (F) lighting. Values are the mean ± SD of six replicates (*n* = 6). The different lowercase letters (a–f) represent significant differences for the microgreen species under different lighting (*p* < 0.05), as determined from a one-way ANOVA for spheroid area and necrosis area, respectively. Associated images showing pro-apoptotic effects (necrosis area) of RD-ES are shown (**B**).

After exposure for 120 h to 40 µL green pea extract, there was a significant increase in the necrotic area (Figure 7A,B) and a concomitant significant decrease in spheroid area compared to the untreated control (time zero). The anti-proliferative and pro-apoptotic effects of green pea extract were comparable for plants cultivated under both LED and fluorescent lighting (Figure 7A). As with the 20 µL volume exposure of spheroids with 40 µL Red Rambo (LED and F), there was a significant increase in the necrotic zone compared to the control (Figure 7A,B). A similar increase in the necrotic area was also noted for rocket (LED and F). Green Pea (LED and F), Red Rambo (LED and F), and rocket (LED and F) were shown to have anti-tumor activity (Figure 7A). However, given the cytotoxic effect on healthy L929 cells (Figure 6A) by green pea (F), Red Rambo, and rocket (LED and F), only the green pea cultivated under LED lighting can be considered relevant at this concentration. At the lower concentration (20 µL), only Red Rambo displayed anti-tumor activity (Figure 5A) with no effect on healthy cells (Figure 4A).

#### 3.3.2. Anti-Proliferative and Pro-Apoptotic Effects of Microgreen Extracts on A673 Sarcoma Cells in 2D Culture and in 3D Spheroids

**Figure 8:**
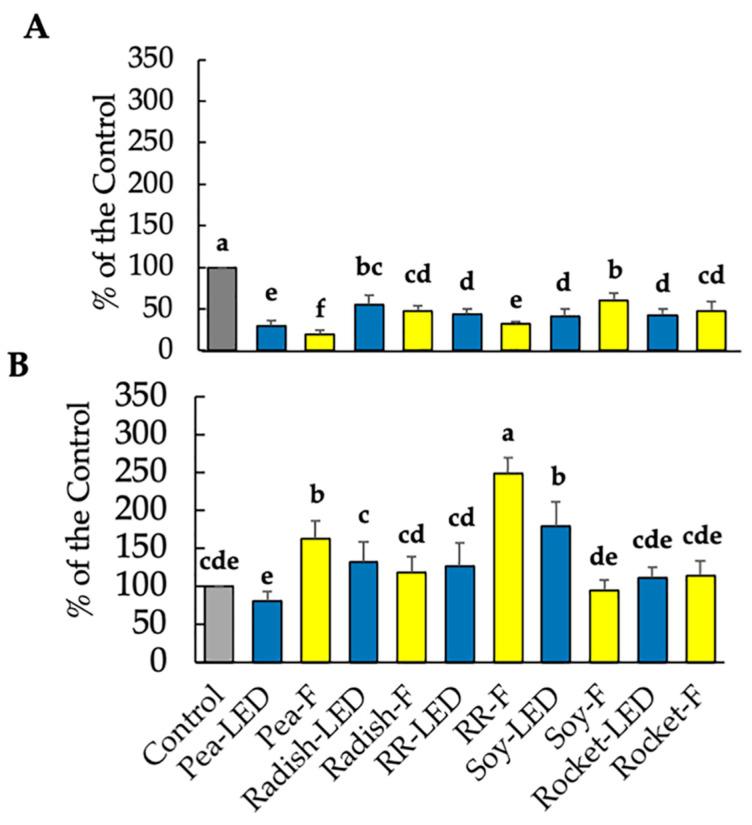
Cell vitality (MTT assay) on 2D A673 (**A**) and 3D A673 (**B**) exposed for 24 h to 20 µL green pea, radish, Red Rambo (RR), soybean (soy), and rocket microgreen extracts, cultivated under LED and fluorescent (F) lighting. Values are the mean ± SD of six replicates (*n* = 6). The different lowercase letters (a–e) represent significant differences between the microgreen species under different lighting (*p* < 0.05) for each treatment, as determined from a one-way ANOVA.

The anti-tumor effects, with specific interest in green pea and Red Rambo, were then examined in morphologically larger spheroid cultures generated from the Ewing sarcoma line A673 (Table S1). The effect of 20 µL microgreen extracts with no cytotoxic effect on L929 fibroblasts, using the MTT test (Figure 4A), was similarly investigated using 2D A673 cells. A significant decrease in cell vitality was reported for all microgreen extracts in 2D A673 cells compared to the control (Figure 8A). This was particularly evident for green pea (LED and F), which produced no effect on the 2D RD-ES cells (Figure 4B). As with the RD-ES (Figure 4C), cell vitality of A673 in 3D tumor spheroid form was not significantly decreased in the presence of the microgreen extracts after 24 h (Figure 8B).

**Figure 9:**
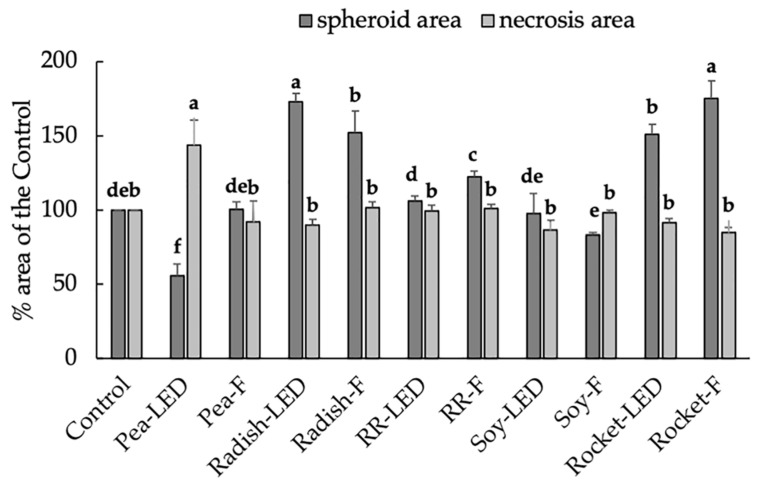
The anti-proliferative (spheroid area) and pro-apoptotic effects (necrosis area) on A673 exposed for 120 h to 40 µL green pea, radish, Red Rambo (RR), soy, and rocket microgreens, cultivated under LED and fluorescent (F) lighting. Values are the mean ± SD of six replicates (*n* = 6). The different lowercase letters (a–e) represent significant differences between the microgreen species under different lighting (*p* < 0.05) for spheroid area and necrosis, respectively, as determined from a one-way ANOVA.

The anti-proliferative (spheroid area) and pro-apoptotic effects (necrosis area) on A673 were then analyzed after 120 h exposure to 20 µL microgreen extracts. The results showed no significant reduction in tumor spheroid area after exposure to any of the microgreen extracts and a small but significant increase in necrosis for green pea (LED) of 111% compared to the 100% of the control (results not shown). Thereafter, the anti-proliferative and pro-apoptotic effects on A673 were analyzed after 120 h exposure to 40 µL microgreen extracts (Figure 9). There was a significant decrease in spheroid area and increased necrosis only in green pea (LED) (Figure 9), which had no negative effect on healthy cells (Figure 7A). There was no anti-tumor effect in the remaining extracts. Interestingly, radish and rocket significantly increased the spheroid area (pro-tumor activity) (Figure 9).

## 4. Discussion

The advent of LED lighting has provided research impetus to examine phytochemical expression in response to light spectra [7,9,10,11,12,13]. Of interest to the present study was the use of combination LED (B:G:R) with a peak spectrum in B wavelength, known to induce increased expression of polyphenols [11,13], with the objective of investigating the functional efficacy of polyphenol constituents extracted from five microgreens (cultivated under fluorescent (control) and LED lighting) on two Ewing sarcoma cell lines. The present study shows, for the first time, the anti-proliferative and pro-apoptotic effects of *Pisum sativum* (LED-grown) microgreen extracts (2.1 µg polyphenols in 40 µL) on both RD-ES and A673 sarcoma in tumor form (3D spheroids), with no cytotoxic effects on healthy L929 fibroblast cells. There was also a noticeable anti-tumor effect of Red Rambo radish microgreen extracts in a 20 µL volume (1.4 and 0.81 µg polyphenols for LED and F, respectively) on only RD-ES sarcoma spheroids without cytotoxic effects on healthy fibroblasts. Though a significant anti-tumor effect was also evident on RD-ES spheroids by Red Rambo radish extracts at the higher extract volume, a cytotoxic effect was also evident on the healthy fibroblasts.

The anti-proliferative and pro-apoptotic effects in 3D were demonstrated by significant decreases in spheroid area attributable to cytotoxic effects on the outer proliferative zone, and increased apoptosis of the central necrotic zone, respectively. Previously, anti-proliferative and pro-oxidant effects of five *Brassicaceae* microgreens were demonstrated on 2D human carcinoma cancer cells [15]. Similarly, in the present investigation, when the RD-ES and A673 sarcoma cell lines were cultivated in 2D, there was a significant anti-proliferative effect to varying degrees after exposure to all microgreen extracts. Collectively, both the present study and that reported previously [15] suggest the dietary efficacy of specific microgreen food matrixes may exercise either a cancer prevention effect (2D cells) or a progression prevention effect (3D spheroids) in the early stages of cancer development.

The anti-proliferative effect by all microgreen extracts on the 2D RD-ES and A673 sarcoma cell lines was evident after 24 h. In contrast, an anti-proliferative effect by relatively few microgreen species (at a higher sample concentration) on the 3D spheroids was only evident after 120 h. Although 2D cultures have proved invaluable in providing knowledge on the functional potential of therapeutic agents, the latter serve as preliminary screening models [32]. With the advent of 3D cancer cell spheroid models, it has been shown that the anti-tumor activity of plant extracts differs in 3D spheroid cancer cell cultures compared to the same cells cultured in a 2D monolayer [33]. A greater resistance to the microgreen extracts was encountered in the more physiologically relevant spheroid cultures, corroborating previous recent findings [34]. The anti-proliferative and pro-apoptotic efficacy on RD-ES and A673 sarcoma spheroids, specifically of green pea (LED-grown) and to some extent, Red Rambo radish (fluorescent-and LED-grown), were hence concluded to have surpassed resistance mechanisms attributable to the physical properties of the spheroid structure. These resistance mechanisms include increasing hypoxia and acidosis gradients from the outer proliferative to the inner necrotic regions, respectively, the deposition of extra-cellular matrix (ECM) proteins, and increased cell–ECM and cell–cell interactions which constitute a physical barrier [19,32].

Noteworthy is that only green pea (LED-grown) had both an anti-proliferative and pro-apoptotic effect on A673 spheroids. None of the remaining microgreens produced an effect, signifying that either the morphology of the A673 spheroids differed (larger) or that different cancer lines differ in resistance mechanisms to the same compounds tested. However, taking into consideration that the same microgreen extracts impacted differently on cell proliferation between RD-ES and A673 in 2D models highlights the need for screening different cancer cell lines when testing for ameliorative effects of food matrixes and phytochemical compounds. Of interest, constraints pertaining to the physical micro-environment of spheroids as well as accompanying epigenetic effects have also been shown to render tumors resistant to cancer drug treatments in clinical settings [19,20,34,35]. Hence, there is the requisite for research into the efficacy of dietary phytochemicals in mitigating constraints related to the physical micro-environment of spheroids and epigenetic modifications [17,20,33,34,35]. This approach may be particularly attractive for rare, hard-to-treat cancer lines such as Ewing sarcoma, displaying resistance mechanisms [21,36], where, although nutraceutical supplementation is sought-after for patients, there is a requisite for phytochemicals with strictly pro-oxidant activity [37].

The present investigation also shows for the first time the anti-tumor efficacy of *Pisum sativum* microgreens. Cancer protection properties of green pea seeds and peels using 2D cell models have been reviewed previously [38]. Regarding leaf material, there is a single report on the cytotoxic activity of recombinant lectins, initially extracted from mature leaf tissue, in MCF-7 (breast) and HepG-2 (liver) cancer lines [39]. In the present study, the heat sterilization procedure would have eliminated any potential contribution of the lectins, which are widely reported to be temperature sensitive. Moreover, the aqueous extraction and sterilization would also have eliminated the contribution of carotenoids and vitamins (ascorbic acid), of which the latter was suggested to have an anti-proliferative effect on Caco-2 cells after exposure to *Brassicaceae* microgreens [15]. In the present study, the anti-tumor effect of green pea was largely limited to the polyphenols in the aqueous food matrix extracts, and as such may be representative of either individual or combinations of polyphenol constituents. Future work, using an approach similar to that developed by de la Fuente et al. (2019) [40] involving ethanol extraction, suspension of dried extracts in water followed by pepsin, and pancreatic digestion to examine the effect of bio-accessible components [15], would be an optimal strategy to examine the effects of additional phytochemical constituents within the food matrix. However, in the present work, the interest was centered on the effect of light quality with a spectral predominance of B, known to induce polyphenol constituents, to investigate potential pro-oxidant effects in mitigating tumor progression.

The results of the present study corroborate previous investigations which demonstrated an increased presence of polyphenols and anti-oxidant activity with B LED [11,12,13]. Of relevance is that different spectra in LED lighting have been shown to induce the expression of different individual polyphenol constituents within the total pool [7,12]. Hence, the anti-tumor effect on green pea A673 spheroids was likely attributable to specific constituents synthesized preferably under LED and not fluorescent lighting. Although anti-proliferative/pro-oxidant activity of green pea (40 µL) was evident on RD-ES spheroids under both light treatments, the fluorescent lighting and not LED produced a cytotoxic effect on L929 fibroblasts. For RD-ES cells, this indicates that whilst the anti-tumor effect was not restricted to polyphenols expressed preferably under LED lighting, an anti-oxidant protective effect on healthy fibroblasts was evident from constituents expressed preferably under LED. Interestingly, in Red Rambo radish, anti-proliferative/pro-oxidant effects on RD-ES spheroids occurred using both a 20 and 40 µL extract volume, irrespective of light treatment and cytotoxic effects on healthy fibroblasts. This shows that anti-tumor effects were attributable to polyphenol constituents sufficiently expressed under both light treatments, despite the significantly overall lower polyphenol content under florescent lighting. Total polyphenol content from all five microgreens was not shown to be related to cytotoxic effects in either the sarcoma lines or healthy fibroblast lines, corroborating a largely species-dependent effect [8,11]. Notwithstanding the higher overall polyphenol content of Red Rambo, followed by soybean under LED, neither species displayed anti-proliferative effects on A673, and soybean was similarly ineffective on RD-ES. Hence, our results implicate the role of specific polyphenol constituents within the total pool and not overall polyphenol content.

The overall polyphenol content of Red Rambo was higher than the two remaining Brassica species (radish and rocket) in the present study, corroborating previous results also showing higher contents, as well as FRAP, under conditions favoring a predominance in B LED light [11]. The total polyphenol content of Red Rambo in the present study, if expressed on a dry mass basis, was comparable with that reported under B light [11]. The polyphenol content of rocket was similarly comparable to that reported previously [8]. The present polyphenol contents of soybean exceeded those reported previously under monochrome B LED light [12]. Although polyphenol contents for green pea microgreens reported previously are comparable to that of the present study [41], to the best of our knowledge there is no information pertaining to the effect of B LED in green pea.

Aside from the potential anti-tumor effect of green pea polyphenols (LED-grown), the latter was shown to be fast growing, producing favorable growth quality parameters (fresh weight, ground cover) under both light treatments within the 10 day period. Reduced hypocotyl length (height) under increased B LED, evident in green pea, was reported previously [13] but is not a quality requisite. The higher light intensity emitted by the LED, compared to that of the fluorescent treatment, may be a contributory factor to the significantly lower chlorophyll content in green pea [13] but was not shown to impact on growth. Red Rambo radish, rich in polyphenol content, also displayed potential anti-tumor effects. Growth quality parameters may be improved in this species by extending cultivation to 15 days [11].

## 5. Conclusions

The results show the importance of testing the functional efficacy of phytochemical extracts on spheroid cultures, which more accurately mimic some features of solid tumors. Whereas all the microgreen extracts reduced cell proliferation of Ewing sarcoma RD-ES and A673 cell lines in 2D, only green pea and, to a lesser extent, Red Rambo radish were effective on 3D tumor spheroids. The present study shows, for the first time, promising anti-proliferative effects of *Pisum sativum* (LED-grown) microgreen extracts (2.1 µg polyphenols in 40 µL) on both RD-ES and A673 sarcoma spheroids, with no cytotoxic effects on healthy L929 fibroblast cells. Of interest for potential anti-tumor effects is also Red Rambo radish, rich in polyphenol content. With the promising effects of LED-grown green pea and Red Rambo, further studies should be expanded to include the ultraviolet (UV) A, B, and C spectrum, also reported to induce polyphenol synthesis [10]. Future work on green pea and Red Rambo radish necessitates assessing anti-tumor effects on cancer spheroids with spectra favoring polyphenol content, but with the inclusion of additional phytochemicals naturally occurring in the food matrix. Although the focus of the present study was on the polyphenol content, the benefits of microgreens as functional edible raw foods necessitate an overall assessment of potential phytochemicals, not performed in this study. For this purpose, we propose an extraction protocol similar to that performed on Brassica microgreens [40].

## Figures and Tables

**Table 1:** Growth parameters for species (green pea, radish, Red Rambo, soybean, and rocket microgreens) and lighting (LED and fluorescence) at 10 days.

	Fresh Weight (g)	Dry Weight (g)	Height (mm)	Ground Cover (%)
Species	***	***	***	***
Pea	13.0 ± 2.1 ^a^	1.2 ± 0.20 ^a^	145 ± 23.2 ^a^	185 ± 43.9 ^a^
Radish	9.2 ± 1.1 ^b^	0.7 ± 0.08 ^b^	95.0 ± 11.0 ^c^	190 ± 56.8 ^a^
Red Rambo	6.3 ± 0.9 ^c^	0.4 ± 0.06 ^b,c^	94.0 ± 6.5 ^c^	127 ± 30.4 ^b^
Soybean	4.9 ± 2.3 ^c^	0.7 ± 0.40 ^b^	130 ± 26.7 ^b^	106 ± 60.5 ^b^
Rocket	2.1 ± 0.5 ^d^	0.1 ± 0.25 ^c^	55.7 ± 3.2 ^c^	63.7 ± 14.8 ^c^
Lighting	ns	ns	***	ns
LED	6.7 ± 3.7	0.7 ± 0.4	91.9 ± 23.6	138 ± 66.9
Fluorescent	7.5 ± 4.5	0.6 ± 0.5	116 ± 41.3	131 ± 63.
Species × Lighting	ns	ns	***	*

Species and Lighting (one-way ANOVA), Species × Lighting (two-way ANOVA). Significance levels for Species, Lighting, and Species × Lighting (ns = not significant * = *p* < 0.05, ** = *p* < 0.01, *** = *p* < 0.001). The different lowercase letters (a–d) within each column denote significant differences for each microgreen species at *p* < 0.001.

**Table 2:** Significance levels of the average relative growth rate (RGR), leaf area index (LAI), and net assimilation rate (NAR) for Species, Lighting, and Species × Lighting over a 10 day period.

	RGR	LAI	NAR
Species	***	***	***
Lighting	ns	ns	ns
Species × Lighting	ns	ns	ns

Species and Lighting (one-way ANOVA), Species × Lighting (two-way ANOVA). ns = not significant * = *p* < 0.05, ** = *p* < 0.01, *** = *p* < 0.001.

**Table 3:** Significance levels of phytochemicals and FRAP for Species, Lighting, and Species × Lighting at 10 days.

	Polyphenols	FRAP	Chlorophyll	Carotenoids
Species (descending order)	*** (RR ^a^, Soy ^b^, Pea ^c^, Radish-Rocket ^d^)	*** (RR ^a^, Radish ^b^, Pea-Soy ^c^, Rocket ^d^)	*** (Pea ^a^, Rocket ^b^, RR-Soy-Radish ^c^)	*** (Radish ^a^, Soy ^b^, RR ^c^, Pea ^d^, Rocket ^e^)
Lighting	*** (LED)	*** (LED)	*** (F)	*** (F)
Species × Lighting	***	***	***	***

Species and Lighting (one-way ANOVA). Letters denote significant differences. Species × Lighting (two-way ANOVA). RR (Red Rambo), soy (soybean). ns = not significant, * = *p* < 0.05, ** = *p* < 0.01, *** = *p* < 0.001. The different lowercase letters in each column (phytochemical and FRAP) denotes significant differences for the microgreen species at *p* < 0.001.

**Table 4:** Total polyphenol content in microgreen extracts and the content present in the sterilized 20 µL extract matrix administered to cell lines.

Plant-Light Source	Polyphenols (µg/gFW)	µg Polyphenols in 20 µL Volume
Green pea-LED	1073 ± 97	1.04 ± 0.07
Green pea-F	641 ± 53	0.67 ± 0.01
Radish-LED	791 ± 43	0.73 ± 0.06
Radish-F	316 ± 4.6	0.32 ± 0.01
Red Rambo-LED	1495 ± 53	1.43 ± 0.07
Red Rambo-F	736 ± 9.2	0.81 ± 0.08
Soybean-LED	1203 ± 14	1.15 ± 0.05
Soybean-F	772 ± 58	0.77 ± 0.06
Rocket-LED	733 ± 17	0.75 ± 0.04
Rocket-F	366 ± 24	0.33 ± 0.01

## Supplementary Materials

The following are available online at https://www.mdpi.com/article/10.3390/foods10081690/s1, Figure S1: Light quality of the florescent (blue) and LED (red) light treatments, Figure S2: Growth and morphology of RD-ES spheroids, Table S1: Spheroid area and diameter of RD-ES cells.

## References

[1] Xiao Z., Lester G.E., Luo Y., Wang Q. (2012). Assessment of Vitamin and Carotenoid Concentrations of Emerging Food Products: Edible Microgreens. J. Agric. Food Chem..

[2] Choe U., Yu L., Wang T.T.Y. (2018). The Science behind Microgreens as an Exciting New Food for the 21st Century. J. Agric. Food Chem..

[3] Kyriacou M.C., Rouphael Y., Di Gioia F., Kyratzis A., Serio F., Renna M., De Pascale S., Santamaria P. (2016). Micro-scale vegetable production and the rise of microgreens. Trends Food Sci. Technol..

[4] Ferrarini L., Pellegrini N., Mazzeo T., Miglio C., Galati S., Milano F., Rossi C., Buschini A. (2011). Anti-proliferative activity and chemoprotective effects towards DNA oxidative damage of fresh and cooked Brassicaceae. Br. J. Nutr..

[5] Pasko P., Bukowska-Strakova K., Gdula-Argasińska J., Tyszka-Czochara M. (2013). Rutabaga (Brassica napus L. var. napobrassica) Seeds, Roots, and Sprouts: A Novel Kind of Food with Antioxidant Properties and Proapoptotic Potential in Hep G2 Hepatoma Cell Line. J. Med. Food.

[6] Le T.N., Chiu C.-H., Hsieh P.-C. (2020). Bioactive Compounds and Bioactivities of *Brassica oleracea* L. var. *Italica* Sprouts and Microgreens: An Updated Overview from a Nutraceutical Perspective. Plants.

[7] Kyriacou M.C., El-Nakhel C., Pannico A., Graziani G., Soteriou G.A., Giordano M., Zarrelli A., Ritieni A., De Pascale S., Rouphael Y. (2019). Genotype-Specific Modulatory Effects of Select Spectral Bandwidths on the Nutritive and Phytochemical Composition of Microgreens. Front. Plant Sci..

[8] Xiao Z., Rausch S.R., Luo Y., Sun J., Yu L., Wang Q., Chen P., Yu L., Stommel J.R. (2019). Microgreens of Brassicaceae: Genetic diversity of phytochemical concentrations and antioxidant capacity. LWT.

[9] Craver J.K., Gerovac J.R., Lopez R.G., Kopsell D.A. (2017). Light Intensity and Light Quality from Sole-source Light-emitting Diodes Impact Phytochemical Concentrations within Brassica Microgreens. J. Am. Soc. Hortic. Sci..

[10] Alrifai O., Hao X., Marcone M.F., Tsao R. (2019). Current Review of the Modulatory Effects of LED Lights on Photosynthesis of Secondary Metabolites and Future Perspectives of Microgreen Vegetables. J. Agric. Food Chem..

[11] Alrifai O., Hao X., Liu R., Lu Z., Marcone M.F., Tsao R. (2020). Amber, red and blue LEDs modulate phenolic contents and antioxidant activities in eight Cruciferous microgreens. J. Food Bioact..

[12] Zhang X., Bian Z., Li S., Chen X., Lu C. (2019). Comparative Analysis of Phenolic Compound Profiles, Antioxidant Capacities, and Expressions of Phenolic Biosynthesis-Related Genes in Soybean Microgreens Grown under Different Light Spectra. J. Agric. Food Chem..

[13] Zhang X., Bian Z., Yuan X., Chen X., Lu C. (2020). A review on the effects of light-emitting diode (LED) light on the nutrients of sprouts and microgreens. Trends Food Sci. Technol..

[14] Calvani M., Pasha A., Favre C. (2020). Nutraceutical Boom in Cancer: Inside the Labyrinth of Reactive Oxygen Species. Int. J. Mol. Sci..

[15] De La Fuente B., López-García G., Máñez V., Alegría A., Barberá R., Cilla A. (2020). Antiproliferative Effect of Bioaccessible Fractions of Four Brassicaceae Microgreens on Human Colon Cancer Cells Linked to Their Phytochemical Composition. Antioxidants.

[16] Koh Y.-C., Ho C.-T., Pan M.-H. (2020). Recent advances in cancer chemoprevention with phytochemicals. J. Food Drug Anal..

[17] Singh V.K., Arora D., Ansari M.I., Sharma P.K. (2019). Phytochemicals based chemopreventive and chemotherapeutic strategies and modern technologies to overcome limitations for better clinical applications. Phytother. Res..

[18] Galieni A., Falcinelli B., Stagnari F., Datti A., Benincasa P. (2020). Sprouts and Microgreens: Trends, Opportunities, and Horizons for Novel Research. Agronomy.

[19] Costa E.C., Moreira A.F., Diogo D.M.D.M., Gaspar V., Carvalho M.P., Correia I.J. (2016). 3D tumor spheroids: An overview on the tools and techniques used for their analysis. Biotechnol. Adv..

[20] Shankar E., Kanwal R., Candamo M., Gupta S. (2016). Dietary phytochemicals as epigenetic modifiers in cancer: Promise and challenges. Semin. Cancer Biol..

[21] Nunes A.S., Barros A.S., Costa E.C., Moreira A.F., Correia I.J. (2019). 3D tumor spheroids as in vitro models to mimic in vivo human solid tumors resistance to therapeutic drugs. Biotechnol. Bioeng..

[22] Singleton V.L., Orthofer R., Lamuela-Raventos R.M. (1999). Analysis of total phenols and other oxidation substrates and antioxidants by means of folin-ciocalteu reagent. Methods Enzymol..

[23] Benzie I.F., Strain J.J. (1996). The Ferric Reducing Ability of Plasma (FRAP) as a Measure of “Antioxidant Power”: The FRAP Assay. Anal. Biochem..

[24] Porra R., Thompson W., Kriedemann P. (1989). Determination of accurate extinction coefficients and simultaneous equations for assaying chlorophylls a and b extracted with four different solvents: Verification of the concentration of chlorophyll standards by atomic absorption spectroscopy. Biochim. Biophys. Acta BBA Bioenerg..

[25] Terrazas-Hernández J., Santos-López E.M., Cariño-Cortés R., Jiménez-Alvarado R., López-Palestina C.U., Hernández-Fuentes A.D. (2018). Effects of Sterilization on Bioactives of Jatropha dioica and Opuntia oligacantha Extracts, and on Antimicrobial Capacity against Streptococcus mutans. Appl. Sci..

[26] Acker H. (1984). Spheroids in Cancer Research. Adv. Struct. Saf. Stud..

[27] Ivascu A., Kubbies M. (2006). Rapid Generation of Single-Tumor Spheroids for High-Throughput Cell Function and Toxicity Analysis. J. Biomol. Screen..

[28] International Organization for Standardization (2009). UNI EN ISO 10993-5:2009, Annex C. MTT Cytotoxicity Test. In Vitro Cytotoxicity Testing.

[29] Truzzi F., Valerii M.C., Tibaldi C., Zhang Y., Abduazizova V., Spisni E., Dinelli G. (2020). Are Supplements Safe? Effects of Gallic and Ferulic Acids on In Vitro Cell Models. Nutrients.

[30] Saltari A., Truzzi F., Quadri M., Lotti R., Palazzo E., Grisendi G., Tiso N., Marconi A., Pincelli C. (2016). CD271 Down-Regulation Promotes Melanoma Progression and Invasion in Three-Dimensional Models and in Zebrafish. J. Investig. Dermatol..

[31] Ho W.Y., Yeap S.K., Ho C.L., Rahim R.A., Alitheen N.B. (2012). Development of Multicellular Tumor Spheroid (MCTS) Culture from Breast Cancer Cell and a High Throughput Screening Method Using the MTT Assay. PLoS ONE.

[32] Białkowska K., Komorowski P., Bryszewska M., Miłowska K. (2020). Spheroids as a Type of Three-Dimensional Cell Cultures—Examples of Methods of Preparation and the Most Important Application. Int. J. Mol. Sci..

[33] Phan N.L.-C., Pham V.P., Nguyen M.T.-T., Phan N.K., Truong K.D., Van Pham P. (2020). Anti-tumor activity of plant extracts against human breast cancer cells are different in monolayer and three-dimensional cell culture screening models: A comparison on 34 extracts. Biomed. Res. Ther..

[34] Schmidt M., Polednik C., Roller J., Hagen R. (2014). Galium verum aqueous extract strongly inhibits the motility of head and neck cancer cell lines and protects mucosal keratinocytes against toxic DNA damage. Oncol. Rep..

[35] Amaral M.V.S., Portilho A.J.D.S., Da Silva E.L., Sales L.D.O., Maués J.H.D.S., De Moraes M.E.A., Moreira-Nunes C.A. (2019). Establishment of Drug-resistant Cell Lines as a Model in Experimental Oncology: A Review. Anticancer. Res..

[36] Riffle S., Pandey R.N., Albert M., Hegde R.S. (2017). Linking hypoxia, DNA damage and proliferation in multicellular tumor spheroids. BMC Cancer.

[37] Favre C. (2019). Antioxidant Nutraceutical approach to Ewing Sarcoma: Where is the Trap?. Biomed. J. Sci. Tech. Res..

[38] Jiraungkoorskul W., Rungruangmaitree R. (2017). Pea, Pisum sativum, and its anticancer activity. Pharmacogn. Rev..

[39] Patel A. (2014). Isolation, characterization and production of a new recombinant lectin protein from leguminous plants. Biochem. Compd..

[40] De La Fuente B., López-García G., Mañez V., Alegría A., Barberá R., Cilla A. (2019). Evaluation of the Bioaccessibility of Antioxidant Bioactive Compounds and Minerals of Four Genotypes of Brassicaceae Microgreens. Foods.

[41] Wojdyło A., Nowicka P., Tkacz K., Turkiewicz I.P. (2020). Sprouts vs. Microgreens as Novel Functional Foods: Variation of Nutritional and Phytochemical Profiles and Their In Vitro Bioactive Properties. Molecues.

