# Supplementary material for: Microgreens: Functional Food with Antiproliferative Cancer Properties Influenced by Light
Source: Foods. 2021 Jul 22;10(8):1690. doi: 10.3390/foods10081690 (PMC8392261; doi:10.3390/foods10081690)
Supplement: Supplementary file 1 [file foods-10-01690-s001.zip › foods-1271312-supplementary material.pdf]

Supplementary Materials

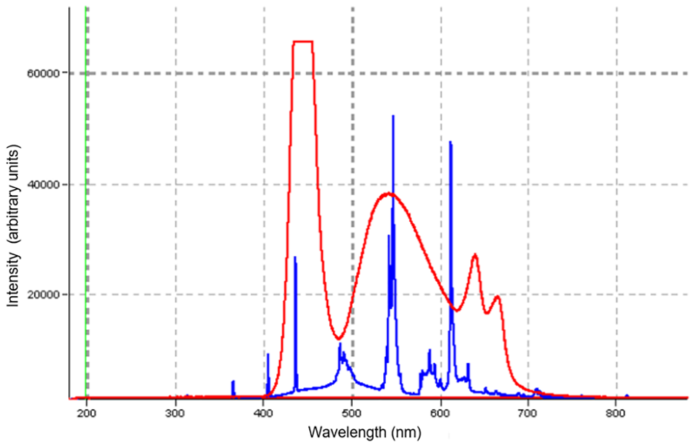

Figure S1. Light quality of the florescent (blue) and LED (red) light treatments. Spectral peaks of the fluorescent lamp show a peak in blue (380-450 nm), with maximum peaks in green (495-570 nm) and orange (590-620 nm). The LED lamp has the maximum emission peak in blue (450-475 nm), and lower peaks in green (495-570 nm), and red (620-700 nm).

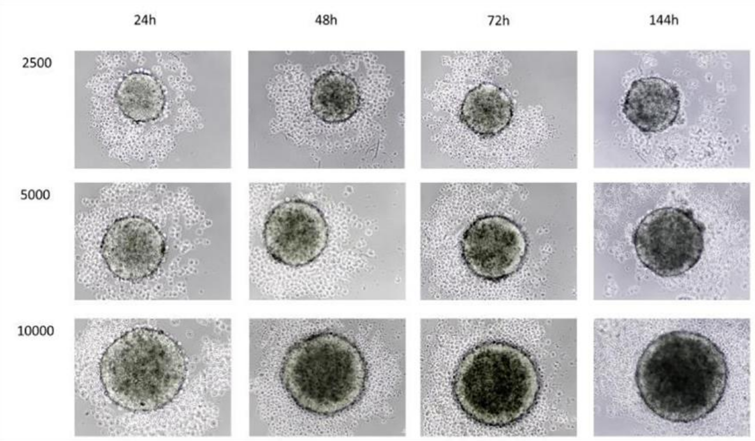

Figure S2. Growth and morphology of RD-ES spheroids generated from 2500, 5000 and 10000 cells at 24, 48, 72 and 144 hours after seeding.

Table S1. Spheroid area and diameter of RD-ES generated from 2500, 5000 and 10000 cells and of A673 generated from 5000 cells after 24, 48, 72 and 144 h.

		RD-ES			
**Cells**	**Measurements**	**24 h**	**48 h**	**72 h**	**144 h**
2500	Area (μm^2)	81205 ± 5230	83191 ± 5682	85013 ± 7801	95560 ± 10882
	Diameter (μm)	321 ± 10.3	325 ± 11.0	329 ± 14.9	348 ± 19.8
5000	Area (μm^2)	137911 ± 8074	145075 ± 5512	152163 ± 4730	176401 ± 10236
	Diameter (μm)	419 ± 12.1	430 ± 8.1	440 ± 6.9	474 ± 13.8
10000	Area (μm^2)	245622 ± 9162	245853 ± 6544	250062 ± 6389	288059 ± 9346
	Diameter (μm)	559 ± 10.5	559 ± 7.5	564 ± 7.2	606 ± 9.9
		**A673**			
5000	Area (μm^2)	186266 ± 20928	210593 ± 8521	227299 ± 14154	342106 ± 15445
	Diameter (μm)	486 ± 27.1	522 ± 10.6	538 ± 16.9	660 ± 14.9
